# Virtual Reality Relaxation for Patients With a Psychiatric Disorder: Crossover Randomized Controlled Trial

**DOI:** 10.2196/17233

**Published:** 2021-01-15

**Authors:** Wim Veling, Bart Lestestuiver, Marieke Jongma, H J Rogier Hoenders, Catheleine van Driel

**Affiliations:** 1 Department of Psychiatry University Medical Center Groningen University of Groningen Groningen Netherlands; 2 VRelax BV Groningen Netherlands; 3 Centre for Integrative Psychiatry Lentis Groningen Netherlands

**Keywords:** virtual reality, stress, relaxation, negative affect, positive affect, depression, anxiety, randomized controlled trial

## Abstract

**Background:**

Virtual reality (VR) relaxation is a promising mental health intervention that may be an effective tool for stress reduction but has hardly been tested in clinical trials with psychiatric patients. We developed an easy-to-use VR self-management relaxation tool (VRelax) with immersive 360° nature videos and interactive animated elements.

**Objective:**

To investigate the immediate effects of VR relaxation on negative and positive affective states and short-term effects on perceived stress and symptoms in patients with a psychiatric disorder, compared to standard relaxation exercises.

**Methods:**

A randomized crossover trial was conducted in 50 patients receiving ambulatory treatment for anxiety, psychotic, depressive, or bipolar disorder. Participants were randomly assigned to start with VRelax or standard relaxation and used both interventions for 10 days at home. They completed 8 visual analog scales of momentary negative and positive affective states before and after each session. Global perceived stress and psychiatric symptoms were measured before and after both intervention periods. Treatment effects were analyzed with multilevel mixed model regression analyses and 2-way analysis of variance.

**Results:**

Both VRelax and standard relaxation exercises led to a statistically significant immediate improvement of all negative and positive affective states. Compared to standard relaxation, VRelax resulted in a significantly greater reduction of total negative affective state (change 16.2% versus 21.2%; t_1684_=−2.02, 95% CI −18.70 to −0.28; *P*=.04). Specifically, VRelax had a stronger beneficial effect on momentary anxiety (t_1684_=−3.24, 95% CI −6.86 to −1.69), sadness (t_1684_=−2.32, 95% CI −6.51 to −0.55), and cheerfulness (t_1684_=2.35, 95% CI 0.51 to 5.75). There were no significant differences between short-term effects of the two treatments on global perceived stress and symptoms.

**Conclusions:**

If the results of this trial are replicated and extended, VRelax may provide a much-needed, effective, easy-to-use self-management relaxation intervention to enhance psychiatric treatments.

**Trial Registration:**

Netherlands Trial Register NTR7294; https://www.trialregister.nl/trial/7096

## Introduction

Stress refers to the physiological, psychological, and behavioral responses to demands or perceived threats that challenge individuals’ resources to manage them [1,2]. This study focuses on the psychological stress response, operationalized as the level of perceived distress and corresponding affective states (eg, feeling anxious, nervous, or down, and less calm, cheerful, or content).

Stress responses are often adaptive but also can increase vulnerability to disease. In psychiatry, stress is a transdiagnostic factor that has been related to both onset, course, and recurrence of mood, anxiety, and psychotic disorders [3,4]. Stress-reducing interventions such as breathing exercises, mental imagery, progressive muscle relaxation, and mindfulness are commonly applied in routine mental health care. These interventions are effective for reducing anxiety, stress, and sleeping problems [5,6], have some effects on level of depressive symptoms [7,8], and have hardly been investigated in patients with psychotic disorders [9]. Another intervention known to reduce stress is exposure to natural environments. Consistent associations have been reported between stress level (physiological and subjective) and time spent in green and blue spaces [10-12]. Negative impact of stress on health may be alleviated by presence of green space [13].

Although stress-reducing interventions may be effective, they are challenging for people with mental health problems. They require initiative, concentration, sustained attention, and energy, which are reduced in many psychiatric disorders, especially in association with exposure to stress [14]. There is a need for easy-to-use stress-reducing interventions that require less effort. Virtual reality (VR) technology offers opportunities to overcome the aforementioned challenges. A combination of visual and auditory stimuli in VR can be used to create an immersive experience that may reduce perceived distress and negative affect without much effort and induce relaxation and positive affect. VR treatments have been developed for various psychological and psychiatric problems, including anxiety and psychosis [15-17]. Most of these VR interventions are therapist-led and include exposure treatment, cognitive behavioral therapy, cognitive training, skills training, and stress management. Over the past decade, several studies have tested stress-reducing VR interventions in clinical and nonclinical samples [17]. A VR program for patients with a stress-related psychiatric disorder showed that confrontation with emotionally charged VR objects improved relaxation and negative mood significantly more than conventional cognitive behavioral therapy [18]. A VR intervention combining roleplays with teaching stress management techniques in a natural VR environment reduced perceived stress in workers with high-stress jobs [19]. A systematic review of VR stress management interventions in the military found 14 small studies, showing that VR stress inoculation decreased perceived stress and negative emotions [20]. Several studies specifically investigated the effect of VR relaxation on stress levels, both in healthy volunteers and people with psychiatric problems, mainly using exposure to VR natural environments [17]. Immersion in VR nature showed improvement of perceived stress, affective responses, and physiological stress measures, to a greater degree than neutral VR environments or two-dimensional nature videos [21-26]. These results suggest that VR-based stress management, in particular immersive VR nature relaxation, is a promising approach for stress reduction that merits further clinical research [17,27]. To the best of our knowledge, no randomized controlled trials investigated the effects of VR nature relaxation as a self-management intervention for stress reduction in patients with a psychiatric disorder. We developed VRelax, a VR self-management relaxation intervention with high-quality 360-degree videos of natural environments and interactive elements that enhance relaxation and focused attention to the environments (see Methods). In this study, we investigated the immediate effects of VRelax on negative and positive affective states of patients receiving outpatient treatment for a psychiatric disorder.

We conducted a crossover randomized controlled trial comparing VRelax with standard relaxation exercises. To evaluate immediate effects, momentary levels of negative and positive affective states were assessed before and after each session. As secondary outcomes, short-term effects (after 10 days) were measured with questionnaires on global perceived stress and psychiatric symptoms (anxiety, depression, paranoia). We hypothesized that (1a) VRelax results in an immediate improvement of negative and positive affective states, (1b) VRelax has a short-term effect on global perceived stress and psychiatric symptoms, and (2) the immediate and short-term effects of VRelax are stronger than the effects of standard relaxation exercises.

## Methods

### Participants

**Figure 1:**
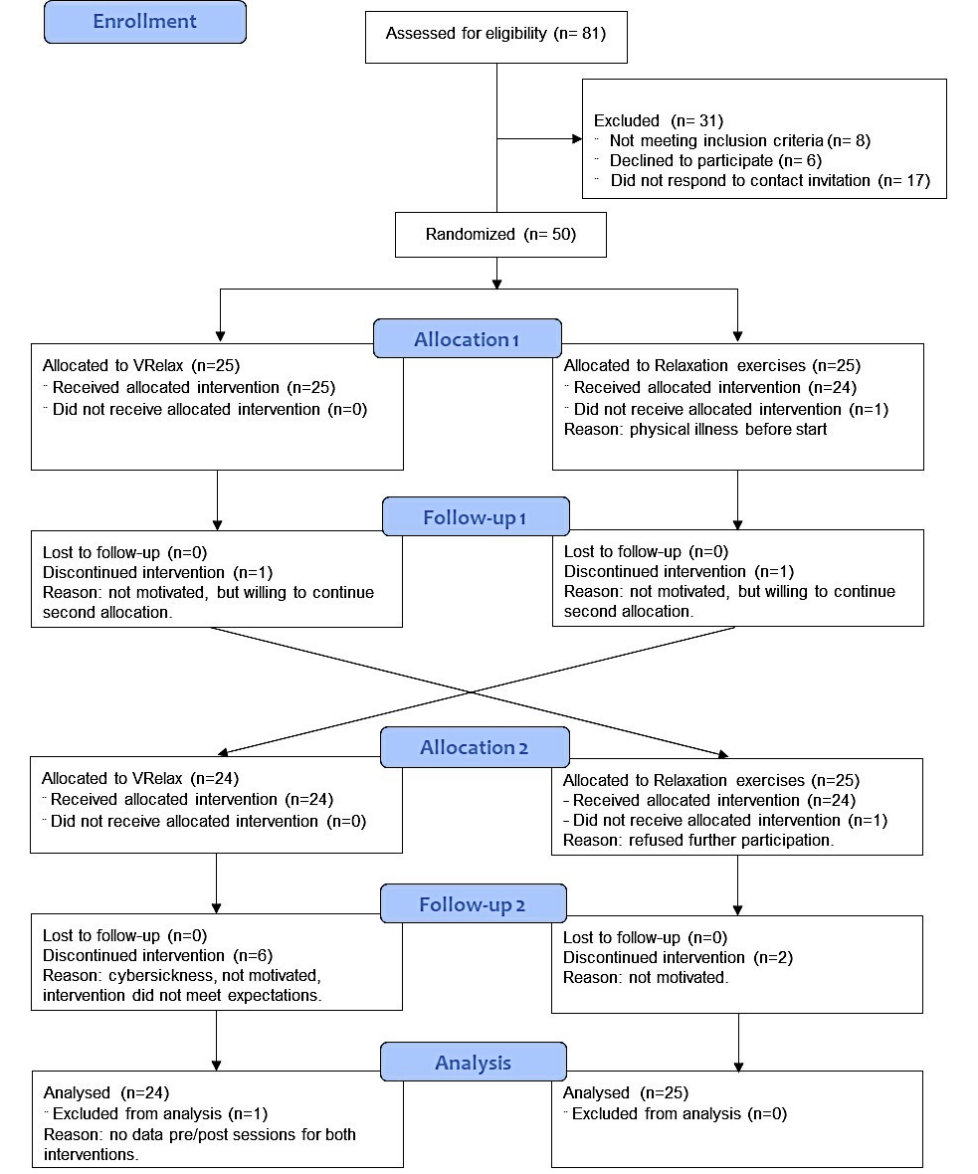
Flowchart of crossover randomized controlled trial.

Patients receiving ambulatory treatment at University Medical Center Groningen (UMCG) Department of Psychiatry, Lentis Center for Integrative Psychiatry, or one of the two participating local general practices were eligible for the study. As stress is a major transdiagnostic factor in psychiatry, and psychiatric disorders often co-occur and partly overlap, a transdiagnostic approach was chosen [28]. Patients could participate if they had a diagnosis of depressive disorder, bipolar disorder, anxiety disorder, or psychotic disorder. Participants were referred to the study by their psychiatrist, psychologist, or general practitioner. A member of the study team contacted potential participants, provided information about the study, checked eligibility, and obtained informed consent. Patients reported their psychiatric diagnosis upon referral to the study; the clinicians who were involved in their regular treatment completed a brief checklist, including diagnosis according to the Diagnostic and Statistical Manual of Mental Disorders, Fifth Edition (DSM-5). The other inclusion criteria were perceived stress, based on self-report or clinician report; older than 18 years; and having a desktop computer or laptop computer at home. Exclusion criteria were a DSM-5 diagnosis of substance use disorder; benzodiazepine use greater than 10 mg per day of diazepam equivalent; diagnosis of epilepsy; and insufficient command of Dutch. A flowchart of the study participants is shown in Figure 1.

### Study Design and Procedures

**Figure 2:**
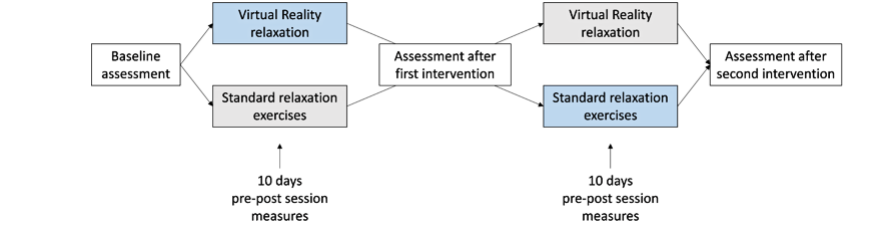
Study procedures.

The study was a randomized controlled crossover trial with two stress-reducing interventions: (1) the VRelax relaxation app and (2) standard relaxation exercises. Study procedures are shown in Figure 2. After informed consent, participants were randomly assigned to either start with VRelax or standard relaxation exercises. Randomization was conducted by an independent UMCG research coordinator using 1:1 randomization, drawing the assignment numbers not in series, but for each participant individually after informed consent was signed. After 10 days, they crossed over to the other intervention. Participants used both interventions consecutively for 10 days at home for a minimum of 10 minutes a day. Negative and positive affective states were assessed before and after each session; other measures were obtained at baseline (T0), after the first intervention period (T1), and after the second intervention period (T2). A crossover design was chosen because patients are very heterogeneous in clinical characteristics and behavior, and many confounding variables can be expected. As the primary outcome was immediate effect of interventions, carry-over effects were expected to be small. Ethical approval was obtained from the UMCG medical ethical committee (protocol number NL64380.042.17). The trial was registered prospectively in the Netherlands Trial Register (number NTR7294).

### Sample Size Calculation

Several studies provided preliminary evidence that VR relaxation reduces psychological and physiological stress, but to the best of our knowledge, no randomized controlled trials investigated the treatment effect of VR relaxation as a separate intervention in patients with a psychiatric disorder. As this was the first randomized VR stress reduction intervention study using VR nature relaxation and an active control condition, there was no estimation of effect size available. The sample size was therefore based on recommendations for a clinical pilot study and determined to be N=50 [29,30].

### Interventions

All patients received regular treatment for their mental health problems throughout the study period. VRelax and standard relaxation were add-on interventions.

#### VRelax

**Figure 3:**
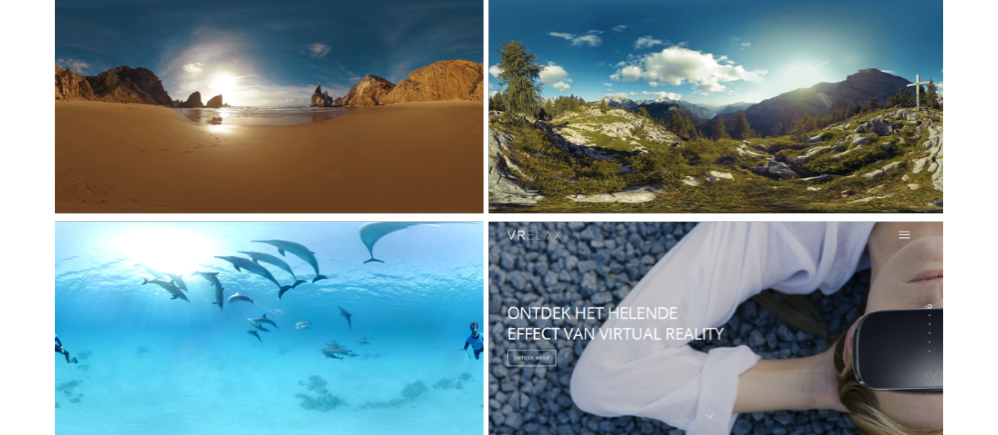
Impression of virtual relaxation environments from VRelax app.

The VRelax tool [31] was used with a Samsung Galaxy S6 or S7 smartphone, connected to a head-mounted display, the Samsung Gear VR. Three-dimensional audio was played with headphones. When VRelax was activated, the participant entered a white-walled waiting room. Presession measures (see Measures section) started automatically within the virtual waiting room. After completion of the presession measures, the walls of the waiting room disappeared, and the participant was standing on a beach, from which he or she could choose where to go. Participants could navigate between interconnected 360-degree video nature environments by looking at hotspots within their field of view that were activated after 2 seconds. VRelax included the following videos: several beaches, a coral reef with tropical fishes, swimming underwater with wild dolphins, a drone flight over a river landscape, a mountain meadow with animals, another mountain scenery in the Alps, a sea view from a cliff, and a beach session of Tibetan singing bowl therapy (Figure 3). Interactive elements were added as an extra layer on top of some videos; for example, audio tracks of guided meditation and progressive muscle relaxation could be activated by looking at hotspots; animated underwater floating air bubbles popped when looking at them; and a pattern of animated circles in the air produced a harmonious melody when looking at them in a particular order.

The 360° videos were created by VIEMR, The Dolphin Swim Club, and Atmosphaeres; the VRelax tool was developed in collaboration with UMCG (first author WV).

Participants received a VRelax set to take home, including Gear VR, smartphone, and headphones. They were completely free to choose which video or videos within the app they preferred to use but were instructed to use the VRelax app at least once daily for 10 minutes. After 10 days, they returned the VR set to the study team.

#### Standard Relaxation

As active control intervention, participants received headphones and a USB stick with audio tracks of guided meditation and progressive relaxation exercises. They could play the tracks on devices they had at home (desktop or laptop computer, or smartphone). They were instructed to use it at least once daily for 10 minutes. After 10 days, participants returned the headphones and USB stick to the study team.

### Measures

#### Primary Outcome

The primary outcome was the immediate effect on level of momentary negative and positive affective states, measured with visual analog scales (VAS; range: 0-100). The VAS items were scored immediately before and after each session, for VRelax within the virtual waiting room, and for the standard relaxation exercises with paper and pencil. Eight ecological momentary assessment items were selected that have been used in previous studies and have been related to psychological stress [32]. The following items were for negative affective states: I feel distressed; anxious; down; nervous (Cronbach α in our sample=.865). The following were for positive affective states: I feel relaxed; calm; cheerful; content (Cronbach α=.897).

#### Secondary Outcomes

Before the first intervention period, after the first intervention period, and after the second intervention period, patients completed questionnaires to assess short-term changes in perceived stress and psychiatric symptoms.

##### Perceived Stress

Global perceived level of stress over the past week was measured with the 10-item version of the Perceived Stress Scale [33], assessing the degree to which situations are appraised as stressful. The Perceived Stress Scale has good psychometric reliability and validity, including in samples of psychiatric patients [34]; in our sample, Cronbach α was .865.

##### Psychiatric Symptoms

Severity of depressive symptoms was measured with the Inventory of Depressive Symptomatology–Self-Report, a widely used 30-item questionnaire with good psychometric characteristics in psychiatric outpatients [35]. Cronbach α was .834 in the study sample. Level of anxiety symptoms was assessed with the 21-item Beck Anxiety Inventory, an instrument with high internal consistency, convergent and discriminant validity [36], and a Cronbach α of .908 in this study. With the Green Paranoid Thoughts Scale, ideas of social reference (16 items) and of persecution (16 items) were assessed [37]. This scale has been developed to assess paranoia over the spectrum of severity and has been recommended as the most reliable and valid self-report measure of paranoia [38]. In our sample, Cronbach α was .982.

#### Other Measures

Demographic and clinical information was obtained from participants: age, gender, marital status, level of education, work situation, DSM-5 classification, illness duration, and psychotropic medication use. During VRelax sessions, how long participants spent in each environment was automatically recorded in log files. Cybersickness was assessed to monitor adverse effects with the self-report Simulator Sickness Questionnaire (SSQ) before and after the VRelax intervention [39].

### Statistical Analyses

All analyses were conducted in SPSS version 26 (IBM Corporation). Analyses of the primary outcome were done by the intention-to-treat approach.

Total negative affective state was calculated by summing the scores of the items anxious, down, distressed, and nervous. Similarly, the items relaxed, calm, cheerful, and content were summed for the total positive affective state. Changes of VAS items and total negative and positive affective state were calculated as 1 – (before session score / after session score). Descriptive statistics (mean, standard deviation, minimum and maximum scores) were calculated for all sociodemographic, clinical, and outcome variables.

As the primary outcome data had a multilevel structure (multiple VAS session scores within participants), VAS scores were analyzed with mixed model multilevel regression analyses (MIXED command), including the factors time (before vs after session), treatment (VRelax compared vs relaxation exercises), order of treatment administration (to adjust for carry-over effects), and the interaction time × treatment. Models had a random intercept for participant and a random slope for order of treatment administration. The estimation method was set to restricted maximum likelihood and the covariance structure to unstructured. First, the immediate effects of interventions were analyzed by comparing mean VAS scores before and after sessions for VRelax and relaxation exercises separately. Second, effects of VRelax compared to relaxation exercises were established by the time × treatment interaction.

Third, 2-way repeated-measures analysis of variance (ANOVA) was used to test differences between VRelax and standard relaxation exercises on global perceived stress, level of psychiatric symptoms, and cybersickness. Finally, carry-over effects were investigated by comparing outcome measures at baseline, T1, and T2 with paired *t* tests (baseline vs T1, T2 vs T1).

## Results

### Overview

Fifty patients were enrolled in this study between March 27 and December 17, 2018. One patient dropped out after randomization and did not start either condition, leaving 49 patients for analyses. For the primary outcome, 1.5% of data was missing (26 of 1762 VAS scores completed by 49 participants). Missing secondary outcome data ranged between 0% and 8.2%. Sociodemographic and clinical characteristics are shown in Table 1.

**Table 1:** Sociodemographic and clinical characteristics of the study sample.

Characteristic		Values
Participants, n		50
Age (years), mean (SD)		41.6 (14.2)
**Sex, n (%)**		
	Male	17 (34)
	Female	33 (66)
**DSM-5 diagnosis, n (%)**		
	Bipolar disorder	13 (26)
	Depressive disorder	16 (32)
	Anxiety disorder	4 (8)
	Psychotic disorder	6 (12)
	Anxiety and depressive disorder	9 (18)
	Anxiety and psychotic disorder	2 (4)
Duration psychiatric illness (years), mean (SD)		9.1 (9.4)
**Marital status, n (%)**		
	Single	22 (44)
	Relationship, not married	10 (20)
	Married	15 (30)
	Divorced	3 (6)
**Level of education, n (%)**		
	Vocational	11 (22)
	Secondary	2 (4)
	Higher	37 (74)
**Medication use, n (%)**		
	Antidepressant	20 (40)
	Antipsychotic	13 (26)
	Mood stabilizer	11 (22)
	Benzodiazepine	17 (34)
	Other psychotropic medication	2 (4)
	No psychotropic medication	9 (18)

The mean number of sessions was 9.4 (SD 3.5; range: 0-16 sessions) for relaxation exercises and 8.7 (SD 4.4; range: 0-24 sessions) for VRelax; the difference was not statistically significant. VRelax log files showed that the average time spent in VRelax video environments was 17 minutes (SD 9.5) per session. Use of different VRelax environments is shown in Table 2. Environments with interactive elements and swimming with wild dolphins were used most frequently.

**Table 2:** Time spent in VRelax, by environment.

VRelax environment	Mean time spent, min:sec	Visits, n	Total time spent, min (%)
Beach with interactive relaxation exercises	8:51	143	1265 (27.7)
Scuba diving with dolphins	5:11	166	862 (18.9)
Sea view from cliff with interactive music element	3:32	136	480 (10.5)
Quiet beach, start position	1:44	275	477 (10.4)
Coral reef with interactive air bubble game	2:39	150	398 (8.7)
Session of Tibetan sound therapy	3:39	88	321 (7.0)
Quiet beach with rocks	1:52	127	238 (5.2)
Mountain scenery without animals	2:17	86	195 (4.3)
Drone flight over river landscape	2:29	67	167 (3.7)
Mountain meadow with cows	2:23	68	162 (3.5)

### Primary Outcome: Immediate Effects

Both VRelax and standard relaxation exercises led to a statistically significant reduction of negative affective states and improvement of positive affective states, as indicated by improvements on all VAS items (Tables 3 and 4). Mean changes in VAS item scores from before to after sessions within individuals were between 11.4%and 28.2% with VRelax, and between 4.6% and 28.3% with relaxation exercises (Table 5). VRelax reduced negative affective states significantly more than relaxation exercises (16.2% versus 21.2%; t_1684_=−2.02, 95% CI −18.70 to −0.28; *P*=.04). Of the separate momentary affective state items, VRelax had a larger beneficial effect than relaxation exercises on feeling anxious, down, or cheerful.

**Table 3:** Effects of VRelax on momentary psychological stress.^a^

VAS^b^ items	Before, mean (SD)	After, mean (SD)	Difference		
			*t*	95% CI	*P* value
Relaxed	44.0 (21.2)	56.4 (22.8)	11.71	10.46 to 14.67	<.001
Calm	44.8 (20.9)	56.1 (23.6)	10.73	9.44 to 13.67	<.001
Cheerful	40.3 (24.9)	44.9 (25.4)	4.92	2.94 to 6.86	<.001
Content	42.2 (23.3)	49.8 (24.8)	8.41	6.00 to 9.65	<.001
Total positive affective state	171.3 (79.0)	207.1 (84.9)	11.11	30.35 to 43.37	<.001
Distressed	45.5 (23.7)	34.7 (22.8)	−9.42	−12.97 to −8.49	<.001
Anxious	31.4 (23.9)	23.8 (22.4)	−7.57	−9.57 to −5.63	<.001
Down	43.5 (24.2)	35.7 (23.2)	−7.10	−10.14 to −5.75	<.001
Nervous	39.2 (24.3)	31.6 (23.1)	−7.32	−9.56 to −5.52	<.001
Total negative affective state	159.6 (79.9)	125.8 (78.2)	−9.65	−40.69 to −26.93	<.001

^a^Mixed model regression analyses, comparison of mean scores after and before sessions, adjusted for order of interventions.

^b^VAS: visual analog scales.

**Table 4:** Effects of relaxation exercises on momentary psychological stress.^a^

VAS^b^ items	Before, mean (SD)	After, mean (SD)	Difference		
			*t*	95% CI	*P* value
Relaxed	44.8 (20.0)	56.8 (19.6)	12.15	10.01 to 13.87	<.001
Calm	42.8 (20.0)	54.9 (19.7)	11.47	9.96 to 14.07	<.001
Cheerful	39.5 (21.8)	41.3 (21.8)	2.19	0.18 to 3.35	.03
Content	41.9 (20.7)	48.2 (20.3)	7.24	4.48 to 7.81	<.001
Total positive affective state	169.0 (72.2)	200.9 (70.2)	10.74	25.94 to 37.54	<.001
Distressed	44.4 (23.5)	34.5 (23.1)	-9.05	−12.13 to −7.80	<.001
Anxious	27.8 (25.3)	24.4 (23.9)	-4.68	−4.81 to −1.97	<.001
Down	40.9 (25.1)	36.6 (24.8)	-4.38	−6.11 to −2.65	<.001
Nervous	36.6 (25.1)	30.0 (23.1)	-7.24	−8.38 to −4.80	<.001
Total negative affective state	149.7 (81.8)	125.4 (81.2)	-9.10	−29.62 to −19.11	<.001

^a^Mixed model regression analyses, comparison of mean scores after and before sessions, adjusted for order of interventions.

^b^VAS: visual analog scales.

**Table 5:** Comparison of treatment effects between VRelax and relaxation exercises.

VAS^a^ items	VRelax change, %^b^	Relaxation exercises change, %	Test differences between interventions^c^			
			*F*	*t*	95% CI	*P* value
Relaxed	28.2	26.8	0.142	0.38	−2.40 to 3.54	.71
Calm	25.2	28.3	0.104	-0.32	−3.52 to 2.53	.75
Cheerful	11.4	4.6	5.510	2.35	0.51 to 5.75	.02
Content	18.0	15.0	0.422	1.24	−0.96 to 4.28	.22
Total positive affective state	20.9	18.9	1.16	1.08	−4.14 to 14.19	.28
Distressed	−23.7	−22.3	0.221	−0.47	−4.04 to 2.48	.64
Anxious	−24.2	−12.2	10.53	−3.24	−6.86 to −1.69	.001
Down	−17.9	−10.5	5.39	−2.32	−6.51 to −0.55	.02
Nervous	−19.4	−18.0	1.537	−0.65	−3.77 to 1.90	.52
Total negative affective state	−21.2	−16.2	4.082	−2.02	−18.70 to −0.28	.04

^a^VAS: visual analog scales.

^b^Percentage of change, mean scores after sessions compared to scores before sessions.

^c^Mixed model regression analyses. Treatment effect is estimated as interaction between time (before/after sessions) and type of intervention, adjusted for order of interventions.

### Secondary Outcomes: Short-term Effects

Short-term effects on perceived stress and symptoms are shown in Table 6. Symptoms of depression and anxiety were reduced significantly after VRelax use. Perceived global stress level, symptoms of depression, and paranoid thoughts were significantly lower after standard relaxation exercises. There were no significant differences between effects of the two treatments, only a trend toward a superior effect of VRelax on anxiety symptoms (interaction time × treatment, *F*_1_=3.650; *P*=.06).

We found period effects on momentary mood states and psychiatric symptoms (Table 7), with stronger effects in the second period than in the first.

No serious adverse events occurred. Several participants reported cybersickness, and 2 stopped using VRelax because of nausea and dizziness. Mean total score on the SSQ, however, was lower rather than higher after VRelax (43.1, SD 10.9) compared to before VRelax (48.3, SD 12.7).

**Table 6:** Perceived stress, psychiatric symptoms, and cybersickness before and after treatments.^a^

Measure	VRelax				Standard relaxation exercises				Difference between treatments^b^	
	Before, mean (SD)	After, mean (SD)	Paired *t* test	*P* value	Before, mean (SD)	After, mean (SD)	Paired *t* test	*P* value	*F* test (*df*)	*P* value
PSS^c^	31.4 (7.5)	30.1 (6.7)	1.65	.11	31.8 (7.3)	29.5 (7.1)	2.77	.01	0.527 (1)	.47
IDS-SR^d^	39.3 (13.6)	35.8 (13.0)	3.31	.002	38.9 (14.4)	36.4 (14.3)	2.34	.02	0.519 (1)	.48
BAI^e^	20.7 (12.3)	16.0 (10.2)	4.30	<.001	18.5 (11.4)	16.8 (12.8)	1.68	.10	3.650 (1)	.06
GPTS^f^	45.8 (21.7)	46.4 (28.1)	0.03	.98	50.1 (29.7)	44.4 (24.1)	2.07	.04	0.760 (1)	.39

^a^Samples sizes of comparisons range between 49 and 45, due to incomplete data.

^b^Two-way ANOVA, interaction term time × treatment.

^c^PSS: Perceived Stress Scale.

^d^IDS-SR: Inventory of Depressive Symptomatology–Self-Report.

^e^BAI: Beck Anxiety Inventory.

^f^GPTS: Green Paranoid Thoughts Scale.

**Table 7:** Changes in momentary affective states, perceived stress and psychiatric symptoms over time.^a^

Measure	T0 (baseline)	T1		T2	
	Mean (SD)	Mean (SD)	*P* value	Mean (SD)	*P* value
Change negative momentary affective states^b^	N/A^c^	−24.7 (43.8)	N/A	−33.2 (38.1)	.003
Change positive momentary affective states^b^	N/A	29.8 (46.7)	N/A	39.6 (40.3)	.001
PSS^d,e^	32.7 (7.5)	30.4 (7.3)	.001	28.8 (6.5)	.22
IDS-SR^e,f^	41.1 (13.7)	36.3 (13.4)	<.001	34.5 (12.5)	.35
BAI^e,g^	21.3 (12.0)	18.0 (11.7)	.002	14.7 (11.2)	.01
GPTS^e,h^	49.8 (27.9)	43.9 (21.0)	.03	45.2 (28.4)	.87

^a^Sample sizes of comparisons range between 49 and 45, due to incomplete data.

^b^Change in mean before/after session scores. T1 and T2 are different subjects because of crossover study design; *P* values for independent *t* tests.

^c^N/A: not applicable.

^d^PSS: Perceived Stress Scale.

^e^Repeated measures; *P* values for paired *t* test, T1 compared to baseline or T2 compared to T1.

^f^IDS-SR: Inventory of Depressive Symptomatology–Self-Report.

^g^BAI: Beck Anxiety Inventory.

^h^GPTS: Green Paranoid Thoughts Scale.

## Discussion

This randomized crossover clinical trial found that VR relaxation immediately reduced negative affective states and improved positive affective states in patients who concurrently received ambulatory psychiatric treatment. VRelax had a stronger effect on negative affective states than standard relaxation exercises, in particular on feeling anxious, down, or cheerful. Psychiatric symptoms, measured over a 10-day period, improved somewhat in both conditions.

The results suggest that immersive VR nature exposure is an effective tool for immediate improvement of affective states and reduction of psychological stress, more powerful than conventional relaxation exercises for making people feel better. The standard relaxation exercises were audio tracks that participants played on their computer or smartphone. Some tracks included 2-dimensional nature pictures. Numerous VR studies have shown that immersion in a virtual environment and the experience of being in a different world strongly induce psychological and physical effects [40]. Although viewing 2-dimensional nature images and videos has previously also been related to stress reduction [41], the immersive nature experience of VR is likely to have contributed to the superior effect.

The immersive VR experience is likely to distract attention from negative thoughts and stimuli, as is found in VR pain distraction studies [42,43]. Attention restoration has been proposed as a key mechanism of health-promoting effects of nature exposure [44-46]. Attention fatigue occurs when, after prolonged and intense use, the capacity to direct voluntary attention is reduced, and the capacity to ward off negative internal or external distractions is reduced [47]. The salient and pleasant characteristics of natural environments capture attention automatically and effortlessly, enabling recovery of voluntary, cognitively directed attention [11,48]. Another potential mechanism originates from psycho-evolutionary theory, stating that exposure to natural environments rapidly restores positive affect and reduces stress-related physiological activation because in earlier times, rapid recovery of stress increased chances of survival [49]. In both theories, nature exposure leads to positive affect and stress reduction, in accordance with the results that were found in the present study.

The effect of VR relaxation was not so strong on global perceived stress over the past 10 days. The perceived stress measure in this study included other aspects of psychological stress besides negative affective states, such as level of perceived control over things that happen, confidence to cope with problems, and ability to overcome difficulties [33]. Whereas affective states are sensitive to immediate and short-term changes [32], the cognitive and behavioral components of stress are likely to change more gradually. Optimal duration of VR relaxation for this purpose may be longer than 10 days, or the study period may have been too short to capture such changes.

Capturing and keeping attention is likely to be more difficult in patients with psychiatric disorders than in healthy individuals because vigilance is impaired and their cognitively directed attention is more severely fatigued [50]. Feedback of psychiatric inpatients during the prototyping phase of VRelax suggested that repeated passive viewing of calm, uneventful virtual nature environments was not always sufficient to distract from negative thoughts and feelings. We therefore added interactive elements to some of the videos, (ie, a relaxation exercise, a simple game of air bubble popping, and musical tones played in response to head movements). Indeed, in this study, the VRelax log files showed that the interactive videos were used longer and more frequently than the “passive” videos.

There was one notable exception: scuba diving with wild dolphins had the highest number of visits. The popularity of the dolphin video can be explained by several factors. Throughout the 7-minute video, there is a lot to be seen all around, with more than 20 active dolphins constantly moving, which may have created a stronger sense of immersion than the other videos. In addition, health-promoting effects on humans have been ascribed to dolphins. Dolphin-assisted therapy is offered for a range of physical and psychological problems, including autism and depression, albeit scientific evidence for efficacy is limited and debated [51,52].

Participants did not report many problems with the use of VR. Only 2 patients stopped using VRelax because of cybersickness, and SSQ scores were low after the intervention. It has been suggested that patients with a psychiatric disorder may experience increased psychiatric symptoms when using VR [16,53]. This may be caused by challenging VR content such as exposure to anxiety-evoking stimuli, which was not part of VRelax. Another potential cause of anxiety and paranoia may be that wearing a head-mounted display disconnects people from their real environment. These issues did not occur during the study, perhaps because participants used VRelax in the safe environment of their own home.

Strengths of this study include the randomized crossover design. Participants served as their own control, minimizing the impact of confounding factors. Analyses of temporal effects suggest carry-over effects of both interventions. Effects on primary and secondary outcomes were stronger in the second intervention period than in the first. However, the impacts of carry-over or period effects on the results of the study are likely to be small because participants were randomized in a 1:1 ratio for order of interventions, and analyses of the primary outcome were adjusted for order of interventions. As a consequence of the crossover design, it was not possible to investigate the effects of VR relaxation on medium- or long-term outcomes. It is conceivable that VR experiences have a longer-lasting impact than standard relaxation exercises because of the immersive character, or simply because people may find it easier to keep using VRelax than to keep doing relaxation exercises. We did not observe such a pattern during the short duration of this study.

Patients used the interventions multiple times over a (double) 10-day period, with effect measures before and after each session. These frequent assessments and detailed longitudinal data increased real life ecological validity and reliability of the primary outcome measure and increased statistical power of the study. A disadvantage was that patients had to reflect on their affective state immediately after relaxation sessions, which may have reduced the beneficial effects to some extent.

Another limitation is that we did not measure physiological effects of VR relaxation, which would have been a valuable outcome measure and a possible way to study biological mechanisms of VR nature exposure. Relaxation therapy, as well as nature exposure, has been related to changes in physiological stress parameters, such as vagal tone, electroencephalography alpha and theta waves, heart rate variability, blood pressure, gene expression, and cortisol level [54,55]. Recent research of VR nature videos found that electroencephalography event-related potentials may be used as a marker of attention restoration [56].

If the results of this trial are replicated and extended, VR nature exposure may provide an effective relaxation intervention to support and enhance psychiatric treatments. Stress management is a cornerstone of psychiatric therapies, but there is a dearth of effortless, pleasant, and powerful self-management tools for improvement of affective states and reduction of stress. Longer-term effects of VR relaxation on perceived stress, psychiatric symptoms, and relapse prevention should be investigated, as well as cost-effectiveness. In addition, integration of biofeedback and more interactive elements (eg, breathing exercises) may increase efficacy of VRelax. Finally, a broader use of VR relaxation can be envisioned for people in need of distraction, for example, during unpleasant medical interventions, during long isolated hospital stays, or for bedridden (terminally) ill patients. VR applications are rapidly growing and may have a profound impact on the landscape of mental health interventions.

## Appendix

Multimedia Appendix 1 CONSORT-eHEALTH checklist (V 1.6.1).

## Abbreviations

ANOVA: analysis of variance
DSM-5: Diagnostic and Statistical Manual of Mental Disorders, Fifth Edition
SSQ: Simulator Sickness Questionnaire
UMCG: University Medical Center Groningen
VAS: visual analog scales
VR: virtual reality
